# Five-Year-Old Sequestered Retained Lens Fragment Requiring Keratoplasty

**DOI:** 10.7759/cureus.9428

**Published:** 2020-07-27

**Authors:** G. Bryant Giles, Donovan Reed, John R Brewer, Charisma Evangelista

**Affiliations:** 1 Ophthalmology, Brooke Army Medical Center, San Antonio, USA; 2 Ophthalmology, Veterans Affairs San Antonio Texas, San Antonio, USA; 3 Ophthalmology, Wilford Hall, San Antonio, USA

**Keywords:** lens fragment, corneal edema, dmek, retained lens fragment

## Abstract

A 77-year-old male with a history of cataract extraction and intraocular lens placement 5.5 years prior, was referred for idiopathic corneal edema of the right eye. Six months prior to initial consult with a Cornea specialist, the patient presented with acute onset cystoid macular edema (CME) and later developed anterior chamber (AC) cell. The cornea became diffusely edematous and decompensated on topical steroids and hypertonic drops. During the Descemet’s membrane endothelial keratoplasty (DMEK) procedure, a sequestered retained lens fragment (RLF) migrated out of the posterior chamber and was aspirated. The remainder of the surgery and post-operative period was unremarkable. This case is the first reported in which a significantly delayed onset of inflammatory reaction from a sequestered RLF led to full corneal decompensation requiring keratoplasty. This case highlights the importance of RLF suspicion in delayed presentation, even when RLFs are not visible via slit-lamp or on gonioscopic view.

## Introduction

Post-operative management of recurrent and/or persistent anterior segment inflammation following cataract surgery presents particular challenges to the ophthalmic surgeon. Causes of prolonged or recurrent post-operative inflammation include retained lens fragments (RLFs), rebound iritis, recurrent uveitis secondary to underlying systemic conditions, and delayed-onset endophthalmitis [1,2]. The diagnostic dilemma may be compounded by a significantly delayed presentation, even years following phacoemulsification.

## Case presentation

A 77-year-old male with a history of adult vitelliform macular dystrophy, mild non-proliferative diabetic retinopathy, mild hypertensive retinopathy, and suspected primary open-angle glaucoma was referred to an academic tertiary care facility for refractory corneal edema. The patient underwent a cataract extraction via bimanual phacoemulsification with a one-piece acrylic intraocular lens placement in the right eye (OD) five years prior to presentation. The surgery was performed by a senior resident and was uncomplicated. Of note, the patient had no history of corneal guttae or other endothelial pathology pre-operatively. He was seen multiple times for post-operative and glaucoma suspect appointments with normal anterior exams. His left eye also had uncomplicated cataract surgery in the same year.

Five years post-operatively, he noted acute onset painless blurry vision in the right eye. His best-corrected visual acuity (BCVA) in the right eye was 20/70 with pin-hole (PH) improvement to 20/40 and intraocular pressure (IOP) of 14. An optical computed tomography (OCT) revealed new cystoid macular edema (CME), which was treated successfully with a one month course of a topical corticosteroid, prednisolone acetate, four times daily and ketorolac tromethamine, a non-steroidal anti-inflammatory drug (NSAID), three times daily. He was subsequently tapered off the anti-inflammatory drop regimen without the recurrence of intra-retinal or sub-retinal fluid. Two months later, he returned with a BCVA of 20/150 (PH 20/100) OD. His intraocular pressure (IOP) was 19 and 16 in his right and left eye, respectively. Slit-lamp examination (SLE) of his right eye was notable for diffuse Descemet’s folds centrally with epithelial edema, central corneal thickness (CCT) of 841um (baseline CCT 566um and 563um two and four years after cataract surgery, respectively) and 2+ anterior chamber (AC) cell. A repeat OCT was performed and demonstrated no retinal pathology. Hypertonic sodium chloride ointment was initiated at four times daily, combination dorzolamide/timolol twice daily, topical corticosteroids four times daily, in addition to a 10-day course of oral acyclovir 400mg five times per day. One week later, he returned with a BCVA of count fingers (CF) at 4 feet, IOP of 9, and CCT of 798um. SLE revealed no AC cell, but diffuse Descemet’s folds, mild stromal edema, and diffuse epithelial edema with some clearing centrally. A bandage contact lens (BCL) was placed in addition to topical fluoroquinolone antibiotic drops and cyclopentolate three times per day. At this time, a referral was placed for a Cornea specialist evaluation for further management recommendations. Over the next two months until his referral appointment, his BCVA ranged from 20/150 to CF at 3 feet with continued corneal decompensation and ultimate development of central bullae.

Of note, his last documented gonioscopic examination, four years prior to presentation revealed open angles to scleral spur 360 degrees without synechiae in both eyes. There were no retained lens fragments noted during gonioscopy.

At his initial consultation with the Cornea service, his exam was unchanged and a Descemet's membrane endothelial keratoplasty (DMEK) was scheduled with plans for an anterior chamber tap for HSV culture and CMV PCR. Prophylactic oral acyclovir at 400mg two times a day was initiated two weeks prior to surgery.

**Figure 1:**
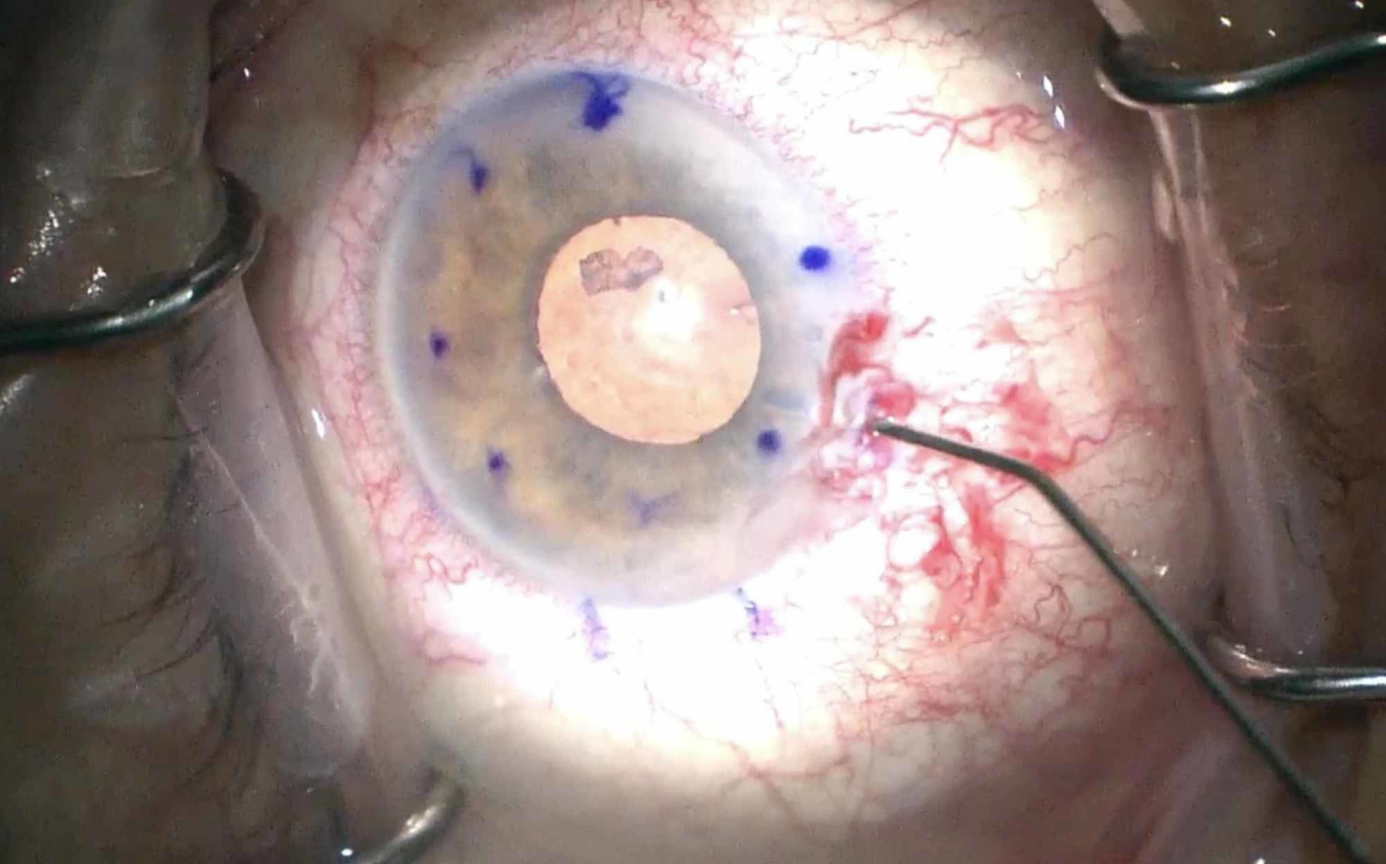
Intraoperative view of retained lens fragment

Intraoperatively, a 1x2mm retained lens fragment presented itself from under the nasal iris, which was subsequently aspirated and successfully extracted from the eye. The DMEK was otherwise uneventful and the patient followed a normal and uncomplicated post-operative course. At the post-operative month one evaluation, the patient’s BCVA was 20/40 with only mild residual microcystic corneal edema peripherally. Viral culture and PCR were negative. Prophylactic dose of oral acyclovir 400mg twice daily was initially continued post-operatively, but discontinued after confirming the viral lab results (Figure 1).

## Discussion

RLFs can incite a prolonged, diffuse inflammatory reaction or local mechanical damage to the corneal endothelium resulting in corneal edema and decreased visual acuity or CME [1,2]. Often, fragment removal leads to reversal of edema and improved vision [3]. The large majority of RLFs are visible in the anterior chamber via slit-lamp; however, some require a gonioscopic view if small and lodged in the anterior chamber angle [2]. RLFs may also remain sequestered behind the iris, only being discovered intraoperatively during anterior chamber washout or after the RLF has migrated into the anterior chamber. Norton and Goyal reported an average time from cataract extraction and RLF removal of 34.7 days [2]. However, few cases of prolonged RLFs have been reported. One case in the literature presented with corneal edema 8.5 years after cataract surgery with a visible inferior angle RLF [4]. Another case was reported to have a visible RLF in the superior capsule 15 years after cataract surgery [5]. In both cases, significant corneal edema was noted with a visible RLF on slit-lamp evaluation, and neither required keratoplasty.

The case presented is the first reported in the literature in which a significantly delayed onset of anterior segment inflammation from a sequestered RLF led to complete corneal decompensation ultimately requiring endothelial keratoplasty.

## Conclusions

This case highlights the importance of clinical suspicion regarding RLFs in delayed presentations of corneal edema and anterior chamber inflammation, even when RLFs are not visible at the slit lamp or during gonioscopy. Despite five asymptomatic years, had a sequestered RLF remained higher on the differential diagnosis, months of prolonged treatment, and corneal decompensation requiring a keratoplasty may have been avoided. Ultrasound biomicroscopy (UBM) may have elicited the culprit had a higher suspicion for RLF been present. As such, anterior chamber washout for possible RFL should be considered in the appropriate clinical context of delayed and unexplained post-operative CME, anterior uveitis, and/or corneal decompensation.
